# Further Characterization of Glycoform-Selective Prions of Variably Protease-Sensitive Prionopathy

**DOI:** 10.3390/pathogens10050513

**Published:** 2021-04-23

**Authors:** Weiguanliu Zhang, Xiangzhu Xiao, Mingxuan Ding, Jue Yuan, Aaron Foutz, Mohammed Moudjou, Tetsuyuki Kitamoto, Jan P. M. Langeveld, Li Cui, Wen-Quan Zou

**Affiliations:** 1 Department of Neurology, The First Hospital of Jilin University, Changchun 130000, China; zwgl18@mails.jlu.edu.cn (W.Z.); dingmx18@mails.jlu.edu.cn (M.D.); 2 Department of Pathology, Case Western Reserve University School of Medicine, Cleveland, OH 44106, USA; xxx13@case.edu (X.X.); jxy43@case.edu (J.Y.); apf28@case.edu (A.F.); 3 Molecular Virology and Immunology Unit (VIM), Université Paris Saclay, INRAE, UVSQ, VIM, 78350 Jouy-en-Josas, France; mohammed.moudjou@inra.fr; 4 Department of Neurological Science, Tohoku University Graduate School of Medicine, 2-1 Seiryo-machi, Aoba-ku, Sendai 980-8575, Miyagi, Japan; kitamoto@med.tohoku.ac.jp; 5 Department of Infection Biology, Wageningen Bioveterinary Research, 8221RA 39 Lelystad, The Netherlands; jan.langeveld@wur.nl; 6 National Prion Disease Pathology Surveillance Center, Case Western Reserve University School of Medicine, Cleveland, OH 44106, USA

**Keywords:** prions disease, Creutzfeldt-Jakob disease (CJD), variably protease-sensitive prionopathy (VPSPr), Gerstmann-Sträussler-Scheinker (GSS), glycoform-selective prion formation, real-time quaking-induced conversion (RT-QuIC) assay

## Abstract

Prion is an infectious protein (PrP^Sc^) that is derived from a cellular glycoprotein (PrP^C^) through a conformational transition and associated with a group of prion diseases in animals and humans. Characterization of proteinase K (PK)-resistant PrP^Sc^ by western blotting has been critical to diagnosis and understanding of prion diseases including Creutzfeldt-Jakob disease (CJD) and Gerstmann-Sträussler-Scheinker (GSS) disease in humans. However, formation as well as biochemical and biological properties of the glycoform-selective PrP^Sc^ in variably protease-sensitive prionopathy (VPSPr) remain poorly understood. Here we reveal that formation of the ladder-like PrP^Sc^ in VPSPr is a PK-dependent two-step process, which is enhanced by basic pH. Two sets of PrP^Sc^ fragments can be identified with antibodies directed against an intermediate or a C-terminal domain of the protein. Moreover, antibodies directed against specific PrP glycoforms reveal faster electrophoretic migrations of PrP fragments mono-glycosylated at residue 181 and 197 in VPSPr than those in sporadic CJD (sCJD). Finally, RT-QuIC assay indicates that PrP^Sc^-seeding activity is lower and its lag time is longer in VPSPr than in sCJD. Our results suggest that the glycoform-selective PrP^Sc^ in VPSPr is associated with altered glycosylation, resulting in different PK-truncation and aggregation seeding activity compared to PrP^Sc^ in sCJD.

## 1. Introduction

Human prion diseases can be sporadic, inherited, or acquired by infection, including Creutzfeldt-Jakob disease (CJD), fatal insomnia, Gerstmann-Sträussler-Scheinker (GSS) disease, kuru, and variant CJD (vCJD). Although they are highly heterogeneous, all these diseases are associated with deposition and accumulation of a pathological and infectious scrapie prion protein termed prion or PrP^Sc^ in the central nervous system [1]. PrP^Sc^ derives from its cellular conformer PrP^C^ through a conformational transition from a α-helix structure into a β-sheet structure [2,3]. The structural transition of PrP^C^ confers altered physicochemical properties to PrP^Sc^ with partial resistance to proteinase K (PK) digestion, insolubility in detergents and propensity to form aggregates. Indeed, comparing PrP^C^ and the PK-resistant PrP^Sc^ (PrP^res^) by western blotting has clearly disclosed that all variably glycosylated PrP^C^ species are able to convert into PrP^res^ in virtually all typical human prion diseases. Moreover, detection and characterization of PrP^res^ fragments by western blotting has significantly improved our understanding of the etiology, pathogenesis, formation of PrP^Sc^, clinical and neuropathological alternations, diagnosis, clinical classification and identification of atypical forms of various human prion diseases [1,4,5,6,7,8,9,10,11,12].

By western blot analysis probing with different PrP-specific antibodies, we previously identified a new human prion strain with a unique electrophoretic profile of PrP^res^ in an atypical human prion disease termed variably protease-sensitive prionopathy (VPSPr) [13,14]. Compared to typical sporadic CJD (sCJD) cases that exhibited approximately 10% PK-sensitive PrP^Sc^, almost 80% PK-sensitive PrP^Sc^ was found in VPSPr homozygous for valine (V) at codon 129 (VPSPr129VV); moreover, strikingly the small amounts of PrP^res^ unveiled a ladder-like electrophoretic profile containing 5 PrP^res^ bands with lower affinity to the widely-used 3F4 antibody but higher affinity to 1E4 [13,14,15,16,17]. Subsequently, we further identified VPSPr in heterozygosity for methionine (M) and V (VPSPr129MV) and in homozygosity for MM at codon 129 (VPSPr129MM); the levels of PrP^res^ were found to be dependent on PrP-129 polymorphism: the highest amounts of PrP^res^ in subjects with VPSPr129MM, followed by VPSPr129MV and VPSPr129VV [14]. Since then, more cases have also been reported by other groups [18,19,20,21,22,23,24,25,26]. Remarkably, using antibodies directed against either PrP mono-glycosylated at residue 181 (Mono181) or at residue 197 (Mono197), besides against the unglycosylated PrP species, we observed for the first time that PrP^Sc^ in VPSPr is derived only from the PrP molecules mono-glycosylated at residue 197 and unglycosylated PrP species, whereas PrP molecules diglycosylated and mono-glycosylated at residue 181 are not converted into PrP^res^ [27,28], a new mechanism underlying prion formation termed “glycoform-selective prion formation”. Notably, VPSPr has been observed to be not transmissible, or with low transmissibility if present [29,30,31]. To date, many questions remain to be addressed about the first reported glycoform-selective prions linked to this new human prion disease. For instance, how is this peculiar electrophoretic profile of PK-induced truncations formed? Why does 1E4 exhibit a higher affinity while 3F4 has a lower affinity to VPSPr PrP^res^ and are there other antibodies that can recognize the ladder-like VPSPr PrP^res^ in addition to 1E4? Are there any differences in PrP glycans between VPSPr and sCJD? Since VPSPr was proposed to be sporadic GSS [14,32,33], is there any difference and similarity between PK-resistant PrP7–8 (a molecular signature of GSS) and VPSPr7? How does the aggregation-seeding activity of VPSPr PrP^Sc^ differ from sCJD prions?

In this study, we address the above questions by antibody mapping of PrP^Sc^ treated with PK and/or PNGase F with one- and two-dimensional western blotting and real-time quaking-induced conversion (RT-QuIC) assay. Our current results demonstrate that VPSPr prions can be further characterized by their peculiar PK-induced truncation procedures and differential antibody binding as well as their lower aggregation-seeding activity compared to sCJD prions.

## 2. Results

### 2.1. Ladder-Like PrP^res^ Electrophoretic Profile Results from PK Dose-Dependent Two-Step Truncation of PrP^Sc^

**Figure 1:**
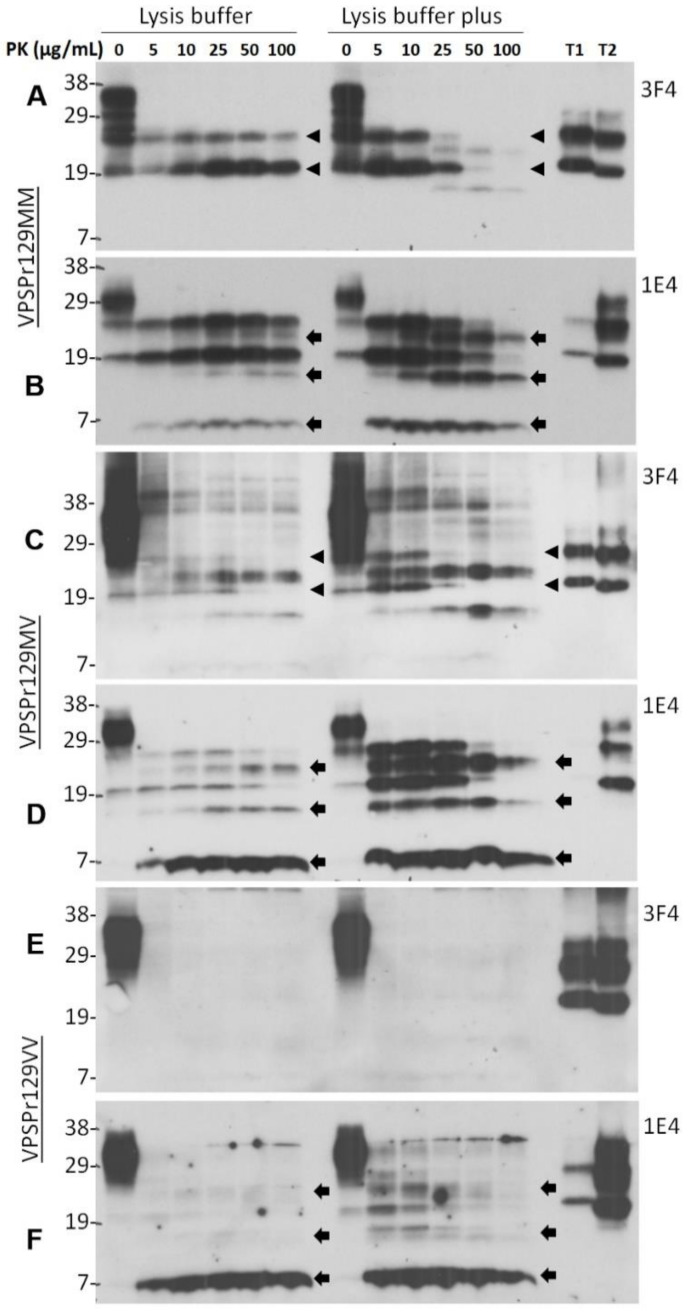
PK concentration-dependent two-step fragmentation of PrP^Sc^ from VPSPr. Brain homogenates from cadavers of VPSPr129MM (**A**,**B**), VPSPr129MV (**C**,**D**), and VPSPr129VV (**E**,**F**), homogenized in standard lysis buffer (pH 7.4) (left sides of western blots) or in lysis buffer “plus” (pH 8.0) (right sides of western blots) were treated with PK at different concentrations ranging from 0, 5, 10, 25, 50, to 100 µg/mL prior to western blotting with 3F4 (**A**,**C**,**E**) or 1E4 (**B**,**D**,**F**). T1: PrP^Sc^ sCJD type 1 control; T2: PrP^Sc^ sCJD type 2 control. Arrow heads indicate ~26 kDa and ~20 kDa PrP^res^ fragments while arrows indicate ~23 kDa, ~17 kDa and ~7 kDa PrP^res^ fragments, respectively.

Our previous studies have discovered that PrP^Sc^ from the brain of patients with VPSPr exhibits, upon PK-digestion, a unique ladder-like electrophoretic profile of 5 PrP^res^ fragments, detectable by the 1E4 antibody but not by the widely-used 3F4 antibody [13,14,15,28,34]. Multiple PK-resistant PrP bands in sCJD could be due to incomplete PK-digestion at lower pH [35,36]. To determine the effect of pH on the generation of the unique PK-resistant PrP fragments and the molecular mechanism underlying the formation of this unique type of PrP^res^, 10% brain homogenate from cadavers of VPSPr129MM, VPSPr129MV, and VPSPr129VV homogenized either in standard “lysis buffer” (pH 7.4) or in “lysis buffer plus” (pH 8.0) were treated with different concentrations of PK from 0, 5, 10, 25, 50, to 100 µg/mL prior to western blotting with 3F4 or 1E4. With the brain homogenates prepared in standard lysis buffer, as shown previously [14,28], 3F4 revealed two PrP^res^ fragments migrating at ~26 kDa and ~20 kDa corresponding to the mono-glycosylated and unglycosylated PrP bands (Figure 1A, left side of the blot, arrow heads); in contrast, 1E4 exhibited three additional bands migrating at ~23 kDa, ~17 kDa and ~7 kDa, in addition to the ~26 kDa and ~20 kDa bands detected by 3F4 (Figure 1B, left side of the blot, arrows). These 1E4-detected PrP^res^ fragments correspond to the N-terminal fragments VPSPr26, 23, 20, 17, and 7 described previously [14,18,28,29,34]. Interestingly, 3F4 also displayed the two additional PrP^res^ bands including VPSPr23 and 17 kDa in the brain homogenates prepared in lysis buffer plus treated with PK at 25–50 µg/mL, although weakly, whereas 1E4 showed increased intensity of the three additional PrP^res^ bands compared to the samples treated with standard lysis buffer at PK concentrations between 5 and 100 µg/mL (Figure 1, right sides of the blots). Notably, the generation of the three bands VPSPr23, 17 and 7 was followed by gradually fading of the two PrP^res^ VPSPr26 and 20. These findings suggest that generation of the unique ladder-like electrophoretic profile of 5 PrP^res^ fragments in VPSPr129MM involves a two-step PK-digestion process: first, generation of mono-glycosylated (VPSPr26) and unglycosylated (VPSPr20) PrP^res^ fragments from the full-length PrP^Sc^ and second, generation of VPSPr23, 17 and 7 from VPSPr26 and 20, respectively. This process can be enhanced at basic pH.

Similar to VPSPr129MM, both VPSPr129MV and 129VV genotypes also revealed the appearance of VPSPr26 and 20 bands first, and the intensity of VPSPr23 and 17 bands increased upon increases in PK-concentration in the samples homogenized with the regular lysis buffer (Figure 1C–F, left sides of the blots). This favors the hypothesis of the two-step process of PrP^res^ generation in VPSPr. Again, more intense ladder-like PK-resistant PrP^res^ was detected in the samples treated with PK in the lysis buffer plus than in regular lysis buffer. Moreover, the affinity of 3F4 to VPSPr PrP^res^ was significantly lower than that of 1E4, especially to VPSPr7 (Figure 1C–F) (Supplemental Table S1). As reported previously [13,14], VPSPr129VV has the least amount of PK-resistant PrP^Sc^ and only 1E4, but 3F4 was able to detect its 5 PrP^res^ fragments (Figure 1E,F). We also noticed that there were some high molecular weight fragments on the 3F4 blots of VPSPr129MV (Figure 1C). There is a possibility that they are dimers or trimers of PrP^res^ based on their molecular sizes.

### 2.2. Two Groups of PrP^res^ Fragments Are Generated upon PK-Treatment of PrP^Sc^

**Figure 2:**
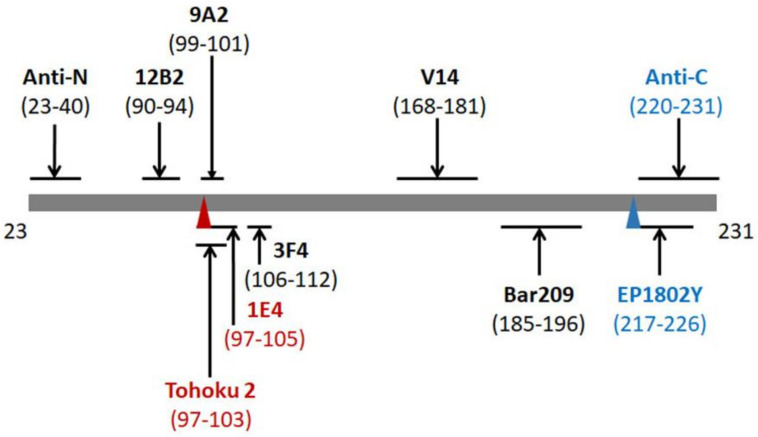
Schematic diagram of epitopes of anti-PrP antibodies used in this study. Epitopes on the mature human full-length PrP sequence from 23–231: anti-N: PrP23–40; 12B2: PrP90–94; Tohoku 2 (T2): PrP97–103; 1E4: PrP97–105; 9A2: PrP99–101; 3F4: PrP106–112; V14: PrP168–181; Bar209: PrP185–196; EP1802Y: PrP217–226; and anti-C: PrP220–231. Red font: antibodies with high affinity to the intermediate fragments VPSPr PrP^res^; blue font: antibodies with high affinity to C-terminal fragments. Red triangle: binding sites for intermediate VPSPr PrP^res^ bands; Blue triangle: binding site for C-terminal VPSPr PrP^res^ bands.

By the combination of the 1E4 antibody with an epitope of PrP97–105 and the anti-C antibody with an epitope of PrP220–231 (Figure 2), our previous study has revealed a total of six different PrP^res^ core fragments in the brain of patients with VPSPr [14]. For instance, after deglycosylation of PrP^res^ with PNGase F, the anti-C antibody detected four deglycosylated PrP^res^ fragments migrating at ~20 kDa, ~18 kDa, ~12/13 kDa, and ~8 kDa, whereas the 1E4 antibody disclosed three deglycosylated PrP fragments VPSPr20, 17 and 7. Of four or three PrP^res^ fragments detected either by anti-C or by 1E4, only the PrP^res^ fragment with the slowest migration (~20 kDa) showed exactly the same molecular weight, suggesting that it contains the epitopes of the two antibodies encompassing PrP97–105 for 1E4 and PrP220–231 for anti-C. Of the rest of the 5 PrP^res^ fragments, three including ~18 kDa, ~12/13 kDa, and ~8 kDa detected by anti-C should have C-terminal sequences all containing the anti-C epitope PrP220–231. In contrast, VPSPr17 and 7 detected by 1E4 should have intermediate PrP sequences all containing the 1E4 epitope PrP97–105 (Figure 2).

**Figure 3:**
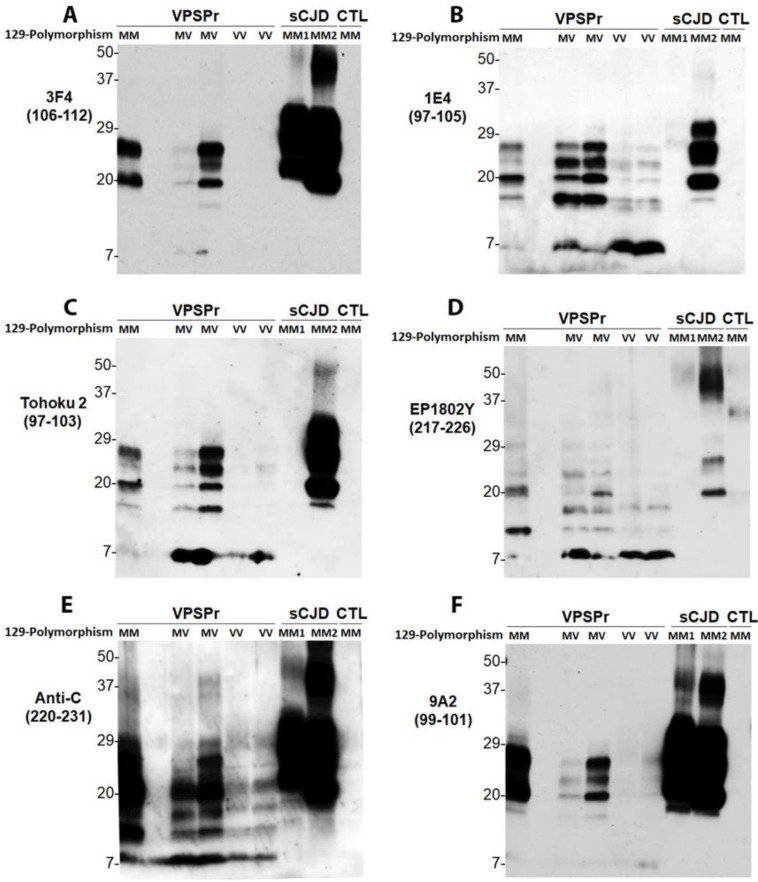
Antibody mapping of PrP^res^ of VPSPr by western blotting. Western blot analysis of PrP in the detergent-insoluble fraction (P2) of the brain homogenates (regular lysis buffer, pH 7.4) of cadavers with VPSPr129MM, VPSPr129MV, or VPSPr129VV after treatment with PK at 50 µg/mL probing with different PrP-specific antibodies. PK-treated PrP in brain homogenates of cadavers with sCJD or non-CJD (CTL) was used as control. Western blotting of PrP^res^ were probed with: (**A**): 3F4 against PrP106–112 [15,39]; (**B**): 1E4 against PrP97–105 [16]; (**C**): Tohoku 2 (T2) antibody against PrP97–103 [37]; (**D**): EP1802Y against PrP217–226 [38]; (**E**): anti-C antibody against PrP220–231 [9]; and (**F**): 9A2 against PrP99–101 [40].

Given that 1E4 but not 3F4 is able to reveal the unique ladder VPSPr PrP^res^ pattern, we hypothesized that only antibodies that have their epitopes with N-terminus starting at residue 97 are able to detect the ladder-like PrP^res^. To test this hypothesis, we probed PrP^res^ without deglycosylation using the Tohoku 2 (T2) antibody that has an epitope PrP97–103 [37] (Figure 2), starting amino acid same as that of 1E4. As 1E4 (Figure 3B), the T2 antibody also exhibited the ladder-like electrophoretic pattern of 5 PrP^res^ fragments, migrating at ~26 kDa, ~23 kDa, ~20 kDa, ~17 kDa, and ~7 kDa (Figure 3C). Regarding the other set of PrP^res^ fragments with the C-terminal regions, we hypothesized that antibodies with epitopes that cover part of the anti-C epitope will be able to disclose C-terminal PrP^res^ fragments of VPSPr. We examined the EP1802Y antibody directed against PrP217–226 [38], overlapping seven out of 12 residues of the anti-C epitope (Figure 2). Like anti-C antibody (Figure 3E), EP1802Y also revealed multiple ladder-like PrP^res^ fragments migrating at ~26 kDa, ~24 kDa, ~20 kDa, ~18 kDa, ~12/13 kDa, and ~8 kDa (Figure 3D). In contrast, the 9A2 antibody with an epitope between residues 99 and 101 (Figure 2) was unable to disclose all PrP^res^ bands (Figure 3F), which behaved more like 3F4 (Figure 3A). The results confirm that the residue 97 as the N-terminal start of the epitope or overlapping with anti-C epitope is the critical to expose the two unique sets of PrP^res^ fragments (Supplemental Table S1). Notably, 3F4, anti-C and 9A2 recognized sCJD PrP^Sc^ MM1 and MM2 equally, whereas 1E4, T2 and EP1802Y exhibited poorer affinity to sCJD PrP^Sc^ MM1 compared to MM2 (Figure 3).

### 2.3. 1E4 Has a Poorer Affinity to the Full-Length but a Better Affinity to Truncated PrP Compared to 3F4

**Figure 4:**
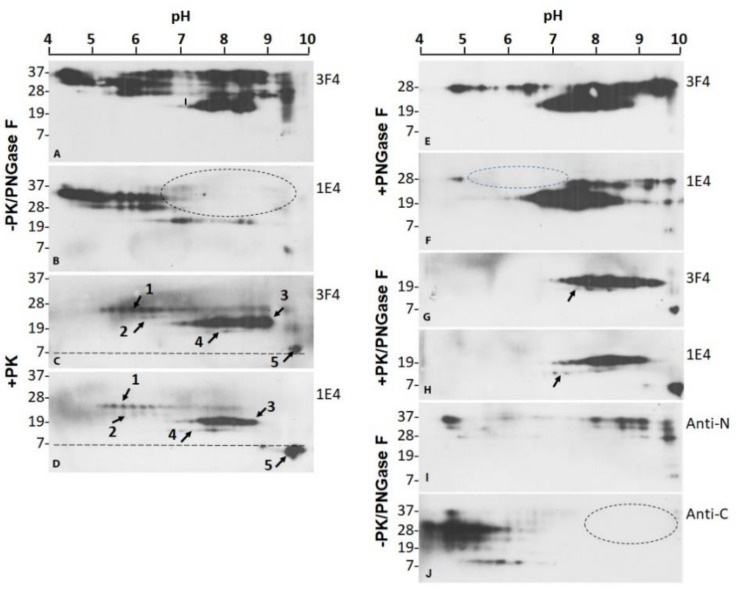
Two-dimensional SDS-PAGE and western blotting of PrP^res^ of VPSPr. Two-dimensional (2D) SDS-PAGE and western blotting of PrP^res^ in brain homogenates of VPSPr was probed with 3F4 (**A**,**C**,**E**,**G**) or 1E4 (**B**,**D**,**F**,**H**) antibodies without treatment (**A**,**B**), treated with PK alone (**C**,**D**), with PNGase F alone (**E**,**F**), and with PK along with PNGase F (**G**,**H**). 2D of PrP from brain homogenates of VPSPr probed with the anti-N (**I**) or anti-C (**J**) antibody without PK and PNGase F treatment. Dotted ovals represent the areas that miss PrP spots on 1E4 or anti-C blots compared to 3F4 or Anti-N antibody.

We further compared the PrP^Sc^ profiles of VPSPr129MV before and after PK and PNGase F treatment by two-dimensional (2D) gel electrophoresis and western blotting probing with 3F4 and 1E4. Without PK and PNGase F treatment, 3F4 detected PrP spots migrating between 37 kDa and 7 kDa within pH range of 4–9.75 (Figure 4A). In contrast, 1E4 displayed PrP spots within pH 4–7.3 but not PrP spots within pH at 7.5–9.75 (Figure 4B, dotted oval), except for the less intense PrP spots migrating at ~20 kDa within pH 6.5–8.8 and ~6–7 kDa with pH 9.5. This finding is consistent with our previous observation and confirmed that 1E4 has a lower affinity to full length PrP but higher affinity to truncated PrP compared to 3F4 [16]. So, the PrP spots with a basic pH that were undetectable by 1E4 correspond most likely to the full-length PrP.

After PK-digestion, both 3F4 and 1E4 antibodies revealed five sets PrP^res^ spots between 28 kDa and 6 kDa within pH 5–9 including three stronger sets (Figure 4C,D, arrows 1, 3, 5) (Supplemental Table S1), corresponding to VPSPr26, 20, and 7, and two weaker sets, corresponding to VPSPr23 and 17 (Figure 4C,D, arrows 2, 4), consistent with our previous findings by one-dimensional (1D) western blotting [13,14,27,28]. However, in contrast to 1D western blotting by which 1E4 but not 3F4 is able to detect VPSPr20, 17 and 7 in the brain homogenates prepared with regular lysis buffer, 2D western blotting showed all five sets of PrP^res^ spots by both 1E4 and 3F4. Furthermore, different from untreated PrP (Figure 4A,B), the PK-treated PrP showed no significant differences in gel profiles between 3F4 and 1E4 blots (Figure 4C,D). Four sets of PrP spots were visible within pH 5.2–9.9, corresponding to VPSPr26 (set 1), VPSPr23 (set 2), VPSPr20 (set 3) and VPSPr17 (set 4), respectively. Notably, PrP spots with the smallest molecular weights (set 5) migrated at 8–7 kDa on the 3F4 blots and at 7–6 kDa on the 1E4 blots despite of the same pH range (Figure 4C,D, arrows 5, dotted lines). In addition, the intensity of VPSPr20 spots was strongest on the 3F4 blots while VPSPr7 was strongest on the 1E4 spots.

After treatment with PNGase F to remove glycans from the protein (without PK), the deglycosylated PrP spots mainly migrated between 29 kDa and 19 kDa within pH 4.5–10 on both 3F4 and 1E4 blots (Figure 4E,F). The two antibodies recognized two major sets of PrP spots corresponding to the deglycosylated full-length PrP at ~29–27 kDa within pH 4.5–10 and truncated PrP at ~26–19 kDa with pH 6.5–8.9 (Figure 4E,F). Again, 1E4 only detected approximately one third of the 3F4-detected deglycosylated full-length PrP spots and virtually all PrP spots within pH 4.0–7.0 were not detected by 1E4 except for a small cluster of spots with pH 4.75 (Figure 4F, dotted oval). Compared to 3F4, 1E4 detected less amount of full-length PrP spots while it recognized more truncated PrP including weaker PrP spots within pH 4.75–5.0 and pH 7–9.8 in addition to the high intense PrP spots within pH 6.0–8.5 (Figure 4E,F). Since the poor affinity of 1E4 to FL-PrP was not changed after deglycosylation, it could be affected by the protein backbone but not the glycans. Moreover, 1E4 detected VPSPr7 that was barely visible on the 3F4 blot.

For PrP treated with PK and PNGase F, both 3F4 and 1E4 displayed two major sets of PrP spots at ~19–22 kDa within pH 7–9.5 and ~7 kDa with pH 10 corresponding to VPSPr20 and 7, respectively (Figure 4G,H) (Supplementary Table S1). VPSPr17 was detected by 1E4 but scarcely detected by 3F4 (Figure 4G,H, arrows). The intensity of VPSPr7 was also higher by 1E4 than by 3F4.

To confirm that the missed PrP spots on the 1E4 blots represent the full-length PrP, we further examined the 2D profile of PrP from VPSPr129MV by antibodies directed against N-terminal domain PrP23–40 (anti-N antibody) and C-terminal domain PrP220–231 (anti-C antibody) [9,41] (Figure 2). For untreated PrP, anti-N detected PrP spots at ~37–26 kDa, corresponding to diglycosylated, mono-glycosylated and non-glycosylated PrP (Figure 4I). These were composed of two major groups of PrP spots: one group within pH 7.3–10 and the other within pH ~4.5–4.8. In contrast, the anti-C antibody recognized PrP spot clusters migrating between ~37 kDa and ~8 kDa within pH 4–6.25 (Figure 4J). No PrP spots were detected in the basic site where there were PrP spots displayed by anti-N (Figure 4J, dotted oval). Therefore, these results confirmed that PrP spots located at the basic side of 2D blot represent the full-length PrP species.

### 2.4. Mono181 and Mono197 PrP Glycoforms May Contain Less or Smaller Glycans in VPSPr Compared to Those in sCJD

VPSPr has been proposed to have abnormal glycosylation in our previous studies [18,28,29,34]. Using antibodies directed against the PrP glycoform mono-glycosylated at residue 181 (Mono181) or 197 (Mono197), we demonstrated that PrP with glycosylation at residue 181 either in diglycosylated or mono-glycosylated forms is not converted into PrP^res^, a phenomenon termed glycoform-selective prion formation [27]. As a result, diglycosylated PrP^res^ and PrP^res^ mono-glycosylated at residue 181 are not detectable in VPSPr.

**Figure 5:**
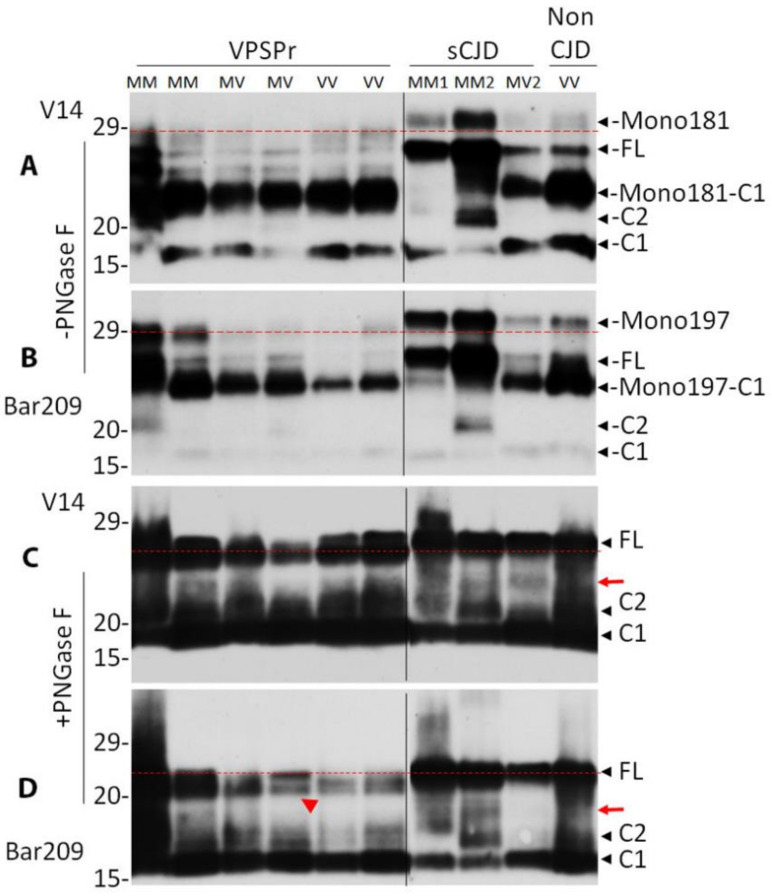
Comparison of PrP glycoforms between VPSPr and sCJD by antibodies with glycan-controlled epitopes. PrP from brain homogenates of VPSPr, sCJD and non-CJD was treated without (**A**,**B**) or with PNGase F (**C**,**D**) prior to western blot analysis probing with antibody V14 (**A**,**C**) or Bar209 (**B**,**D**) that recognizes either PrP mono-glycosylated at residue 181 (Mono181) or residue 197 (Mono197), respectively, while both of them also recognize unglycosylated PrP species. FL: full-length PrP; Mono181-C1: truncated PrP C1 fragment glycosylated at residue 181; Mono197-C1: truncated PrP C1 fragment glycosylated at residue 197; C1: C-terminally truncated PrP fragment migrating at ~18 kDa; C2: C-terminally truncated PrP fragment migrating at ~20 kDa. The red dotted lines are used as a reference for comparison of the top bands in each panel. The red arrows indicate additional band between FL and C2 in sCJD in panels (**C**) and (**D**). The red arrow head indicates the doublets of FL bands in panel (**D**).

To determine whether there are any differences in PrP glycoforms between VPSPr and sCJD before PK-treatment, we next compared their untreated PrP using the two unique antibodies V14 and Bar209 that specifically recognize Mono181 or Mono197, respectively, as well as the unglycosylated PrP [27,42] (Figure 2). As shown previously [27], without PNGase F treatment, 5 PrP bands were detected by the V14 antibody in sCJD and non-CJD brain homogenates from the top to the bottom of the blot: Mono181, unglycosylated full-length PrP (FL), C-terminally truncated PrP fragment mono-glycosylated at residue 181 (Mono181-C1), C-terminally truncated PrP fragment 2 (C2), and C-terminally truncated PrP fragment 1 (C1) (Figure 5A) (Supplemental Table S1). In contrast, V14 showed different PrP banding pattern in VPSPr brain homogenates except for the Mono181-C1 and C1 (Figure 5A). Specifically, VPSPr exhibited no PrP bands that could match the Mono181 band in sCJD and non-CJD (Figure 5A, above the red dotted line). In addition, there were 3–4 PrP bands between Mono181 and Mono181-C1, one of which matched FL in sCJD or non-CJD samples. Since the migration of 1–2 bands were slower than that of FL but faster than Mono181, it is most likely that they are Mono181 but with lower molecular weights compared to that in sCJD.

Bar209 was able to recognize 5 PrP bands as did V14 including Mono197, unglycosylated FL-PrP, C-terminally truncated PrP fragment mono-glycosylated at residue 197 (Mono197-C1), C-terminally truncated PrP fragment 2 (C2) and fragment 1 (C1) (Figure 5B) (Supplementary Table S1). All bands can match between VPSPr and sCJD except for Mono197. The migration of the top PrP band in VPSPr was faster than that of the top Mono197 in sCJD or non-CJD (Figure 5B), suggesting that the Mono197 in VPSPr may have a shorter PrP band or smaller glycans compared to sCJD or non-CJD.

To further determine whether the differences in PrP Mono181 or Mono197 between VPSPr and sCJD or non-CJD result from glycosylation, we examined deglycosylated PrP after treatment with PNGase F probing with V14 and Bar209. Both V14 and Bar209 displayed three major PrP bands in VPSPr, sCJD and non-CJD, corresponding to the deglycosylated FL-PrP, C2 and C1 (Figure 5C,D). There was additional band between the FL and C2 in 3 sCJD cases on the V14 blot and 1 sCJD case on the Bar209 blot (Figure 5C,D, red arrows). It is worth noting that the migration of the top PrP band corresponding to the FL-PrP in sCJD or non-CJD was slightly slower than that of the top PrP band in VPSPr, but the difference was much smaller than that found in glycosylated PrP (Figure 5C,D vs. Figure 5A,B). However, it was noticed that the top band in VPSPr was a doublet while it was a single band in sCJD (Figure 5D, red arrow head).

Taken together, our results suggest that PrP in VPSPr may have an altered glycosylation that results in glycans with smaller molecular weights compared to that in PrP^Sc^ of sCJD, and PrP in VPSPr may have a small N-terminal or C-terminal truncation generating a PrP band that migrated slightly faster than that of FL-PrP.

### 2.5. VPSPr7 Is Different from GSS PrP7–8 and PrP^P102L^ Mutation Inhibits Binding of 1E4 to the Mutant PrP

The PK-resistant intermediate PrP7–8 fragment is the molecular signature of GSS [4,8,43], whose molecular weight is close to that of VPSPr7. To further investigate the banding pattern of PrP^res^ between the two diseases, we treated the brain homogenates from GSS^P102L^-129M and VPSPr129MV with different amounts of PK ranging from 0, 5, 10, 25, 50, 100, and 200 µg/mL, followed by treatment with or without PNGase F. The treated PrP was examined by western blotting probing with different PrP-specific antibodies including 3F4, 1E4, 9A2, 12B2, and anti-C that cover different areas of the protein (Figure 2).

**Figure 6:**
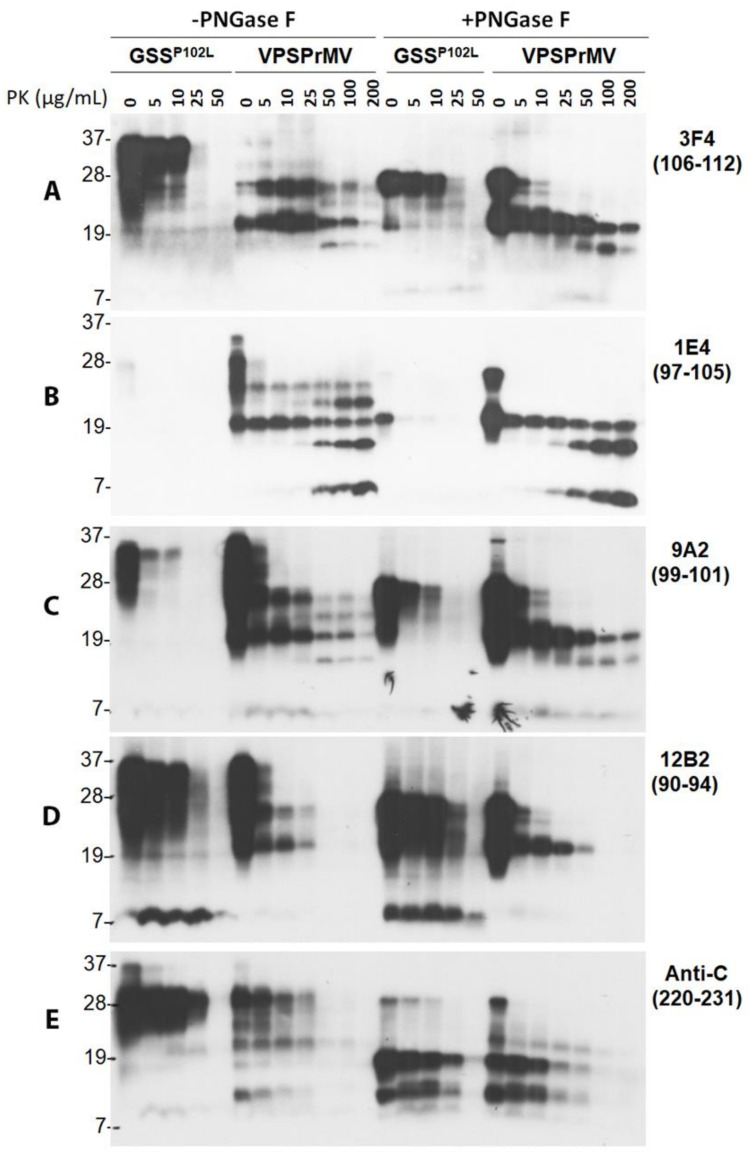
Comparison of electrophoretic profiles of PrP from VPSPr and GSS by western blotting with different PrP-specific antibodies after treatment with different amounts of PK along with or without PNGase F. Brain homogenates from cadavers with GSS^P102L^ or VPSPr129MV were treated with different amounts of PK ranging from 0, 5, 10, 25, and 50 for GSS and 0, 5, 10, 25, 50, 100, and 200 µg/mL for VPSPr129MV at 37 °C for 1 h followed by deglycosylation with PNGase F prior to western blotting probing with different PrP-specific antibodies. (**A**): 3F4 with PrP epitope 106–112 [15,39]; (**B**): 1E4 with PrP epitope 97–105 [16]; (**C**): 9A2 with epitope 99–101 [40]; (**D**): 12B2 with epitope 90–94 [40]; (**E**): Anti-C with epitope 220–231 [9].

On the blot, probing with 3F4, GSS^P102L^ showed no typical PrP^res^ and also no PrP7–8 fragment; instead, there were significant amounts of undigested PrP bands until PK of 10 µg/mL (Figure 6A). After PNGase F treatment, the dominant PrP was the deglycosylated FL-PrP that was not completed digested until 50 µg/mL. A faint band corresponding to PrP7–8 was barely visible. In contrast, PrP from VPSPr before PNGase F treatment exhibited two major PrP bands corresponding to VPSPr26 and 20 until PK of 25 µg/mL. While the intensity of the two bands decreased over increased amounts of PK, two additional bands corresponding to VPSPr23 and 17 were generated at PK of 50 µg/mL or higher (Figure 6A). After PNGase F treatment, there were two major bands in the sample without PK, corresponding to the deglycosylated FL and truncated PrP. Upon increased PK-treatment, the FL-PrP band was completely digested at PK of 25 µg/mL or higher, while the intensity of the truncated PrP was gradually decreased over the increase in the amounts of PK and still detectable at PK of 200 µg/mL. Moreover, VPSPr17 became detectable at PK of 25 µg/mL and reached the highest intensity at PK of 100 µg/mL (Figure 6A).

On the blot with 1E4, virtually no PrP was detected from GSS^P102L^ except for a very faint band migrating at ~28 kDa in the untreated sample and an intense band migrating at ~20 kDa in the sample treated with PNGase F but without PK (Figure 6B), consistent with our previous finding [27]. Since the PrP^P102L^ mutation happens to be in the middle of the 1E4 epitope, it is most likely that this mutation disrupts the binding of 1E4 to the mutant PrP. However, without PNGase F treatment, VPSPr showed detectable VPSPr26 and 20 at every PK concentration examined and additional VPSPr23 and 17 at PK of 25 µg/mL or higher, and 7 at PK of 50 µg/mL or higher (Figure 6B). After PNGase F treatment, VPSPr20 was detectable at every PK-concentration examined while VPSPr17 and 7 became detectable at PK of 25 µg/mL or 10 µg/mL and their intensity was increased more than the increase in the concentration of PK (Figure 6B).

On the blot probing with 9A2, the electrophoretic profile of PrP^res^ from both GSS^P102L^ and VPSPr was similar to that displayed by 3F4 except for showing more intense PrP in the samples without PK-treatment for VPSPr and slightly more intense VPSPr7 than 3F4 (Figure 6C). Notably, similar to 3F4 but different from 1E4, 9A2 revealed that the intensity of VPSPr17 and 7 decreased more than the increase in PK concentration.

On the 12B2 blot, GSS had no typical PrP^res^ in both PNGase F treated and untreated samples except for an intense PrP7–8 (Figure 6D). Moreover, no typical VPSPr PrP^res^ bands were detectable at PK of 50 µg/mL or higher (Figure 6D).

On the blot probing with anti-C without PNGase F treatment, GSS had no typical PrP^res^ except for undigested PrP and a weak PrP^res^ migrating at 8–10 kDa. Treatment with PK and PNGase F generated weak PrP^res^ bands migrating at ~18 kDa, ~12/13 kDa, and ~8–10 kDa, respectively (Figure 6E). Without PNGase F, VPSPr exhibited 5 PrP^res^ bands whose intensity was decreased over an increase in PK-concentration and became barely detectable at PK of 50 µg/mL or higher. As reported previously [14], there were 5 PrP^res^ bands migrating at ~20 kDa, ~18 kDa, ~12 kDa, and 8–10 kDa after PNGase F treatment (Figure 6E).

Therefore, we observed that PrP7–8 in GSS^P102L^-129M is different from that observed in VPSPr cases; it also had the highest affinity to 12B2 while VPSPr7 exhibited the highest affinity to 1E4. Moreover, the GSS^P102L^ mutation dramatically inhibits the affinity of 1E4 to the mutant PrP, most likely because the mutation falls within their epitopes (Figure 2, Supplemental Table S1).

### 2.6. PrP^Sc^-Seeding Activity Is Lower in VPSPr Than in sCJD

**Figure 7:**
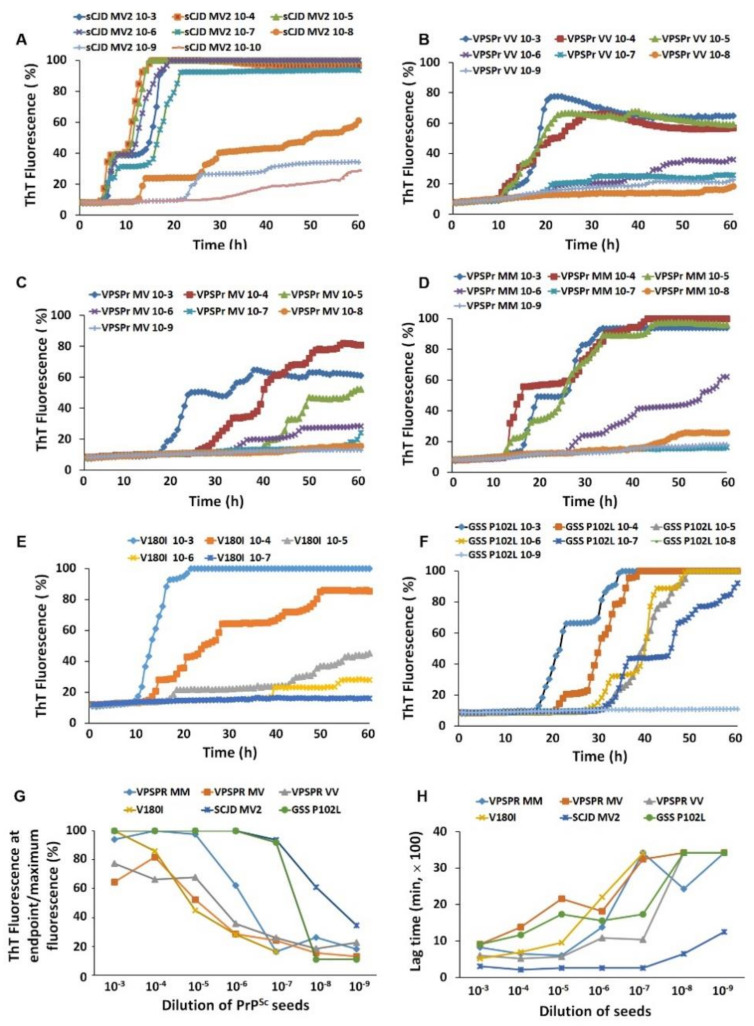
Comparison of brain prion-seeding activity between VPSPr and other prion diseases by RT-QuIC assay. PrP^Sc^-seeding activity was detected with brain homogenates of VPSPr129MM, VPSPr129MV, VPSPr129VV, fCJD^V180I^, GSS^P102L^, or sCJDMV2 as a seed by RT-QuIC assay with recombinant hamster PrP90–231 as a substrate. (**A**): sCJDMV2 (*n* = 1, repeating three times); (**B**): VPSPr129VV (*n* = 3); (**C**): VPSPr129MV (*n* = 3); (**D**): VPSPr129MM (*n* = 2); (**E**): fCJD^V180I^ (*n* = 3); (**F**): GSS^P102L^ (*n* = 3). (**G**): Maximal ThT fluorescence at endpoint of PrP^Sc^ from sCJDMV2, VPSPr129MM, VPSPr129MV, VPSPr129VV, fCJD^V180I^, and GSS^P102L^ as RT-QuIC seeds. (**H**): Lag time of PrP^Sc^-seeding activity with brain homogenates from sCJDMV2, VPSPr129MM, VPSPr129MV, VPSPr129VV, fCJD^V180I^, and GSS^P102L^ as RT-QuIC seeds.

It has been shown that the infectivity of VPSPr is lower, if present, compared to that of sCJD [29,30,31]. To compare aggregation seeding activity of PrP^Sc^ of VPSPr with that of PrP^Sc^ from other more common sporadic and familial prion diseases, we conducted a RT-QuIC assay of serial dilutions of brain homogenate seeds with three genotypes of VPSPr, sCJDMV2 and GSS linked to PrP^P102L^ mutation. We also examined seeding activity of fCJD^V180I^ as another control since fCJD^V180I^ exhibited a similar PrP^Sc^ electrophoretic profile to VPSPr [17,27,28,34]. Although the PrP^Sc^ ThT aggregation fluorescent intensity of sCJDMV2 brain homogenates was significantly decreased until 10^−8^ dilution, weak ThT fluorescent reaction was still detectable as low as 10^−10^ (Figure 7A), consistent with our previous observations [44]. In contrast, of the three genotypes of VPSPr cases, while the PrP^Sc^-fluorescence from VPSPr129VV and 129MV only reached approximately 80% of maximal fluorescence even at the lowest dilutions of 10^−3^–10^−4^, the PrP^Sc^-ThT fluorescence from VPSPr129MM could reach the plateau after 40 h at dilution of 10^−3^ to 10^−5^ and significantly decreased after 10^−6^ dilution (Figure 7B–D). Although the PrP^Sc^ ThT fluorescence at dilution of 10^−3^ reached plateau, it was greatly decreased at dilutions of 10^−4^–10^−5^ and became undetectable after dilution of 10^−6^ in fCJD^V180I^ (Figure 7E). The seeding activity of PrP^Sc^ from GSS^P102L^ was detectable in the brain homogenate diluted from 10^−3^ to 10^−7^ and was not detectable after 10^−8^ and high dilution (Figure 7F). Their log SD50 per milligram of tissues were >8.6 in sCJDMV2, >7.7 in GSS^P102L^, >7.1 in VPSPr129VV, 7.0 in VPSPr129MM, 6.1 in VPSPr129MV, and >5.7 in fCJD^V180I^ (Figure 7G). The PrP^Sc^ seeding activity of sCJDMV2 and GSS^P102^ was significant higher than that of VPSPr129MM, VPSPr129MV, VPSPr129VV, and fCJD^V180I^ in the samples diluted at 10^−7^ (*p* < 0.01) (Figure 7G). In terms of lag time of PrP^Sc^-seeding activity at 10^−6^ dilution, we observed that the sCJDMV2 had the shortest lag time of 4.3 h, followed by 18.0 h for VPSPr129VV, 23.1 h for VPSPr129MM, 26.0 h for GSS^P102L^, 31.1 h for VPSPr129MV, and 36.7 h for fCJD^V180I^ (Figure 7H) (Supplementary Table S1). The lag time of PrP^Sc^-seeding activity at 10^−8^ dilution was significantly shorter in sCJDMV2 than in all other prion diseases examined (*p* < 0.01) (Figure 7H). In sum, PrP^Sc^-seeding activity was lower and its lag time was longer in VPSPr than in sCJD and GSS, consistent with previous findings by RT-QuIC assay and animal transmission studies [24,29,30,31,44].

## 3. Discussion

VPSPr was first identified in 11 subjects homozygous for V at PrP codon 129 [13] and then was found to completely cover the three genotypes by displaying an additional 15 cases heterozygous for M and V as well as homozygous for M [14]. The most striking feature, i.e., the molecular signature of VPSPr, is the universal presence of the ladder-like electrophoretic profile of 5 PrP^res^ fragments. These exhibit unique higher affinity to 1E4 but lower affinity to 3F4 in the brain of all three genotypes of VPSPr, although intensity of the PrP^Sc^ bands is polymorphism-dependent [14]. The classic three PrP^res^ fragments detected in all typical sCJD cases [7,10] are well known to convert from their three counterparts of PrP^C^, including the diglycosylated, mono-glycosylated (Mono181 and Mono197) and unglycosylated PrP species. However, PrP^Sc^ from VPSPr always lacks the diglycosylated PrP while no mutations in human prion protein gene have been identified in all VPSPr cases reported to date [13,14,18,19,20,21,22,23,24,25,26,28]. Remarkably, PrP^res^ in sporadic VPSPr along with fCJD^V180I^ has been further observed to specifically lack not only diglycosylated PrP, but also the PrP Mono181 [27,28]. So far, these have been the only known two prion diseases that have a unique PrP^res^ deriving selectively from Mono197 and non-glycosylated PrP^C^ [17,27,28,34].

**Figure 8:**
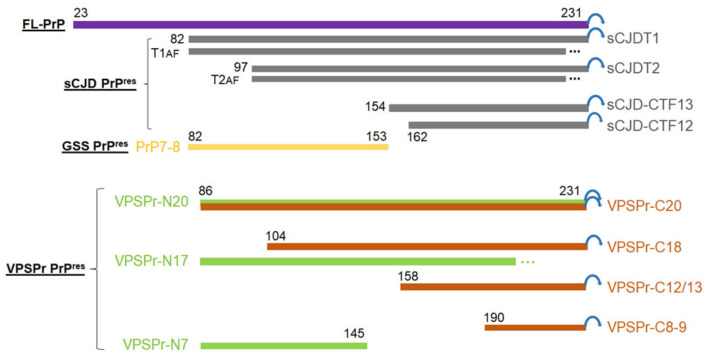
Schematic diagram of various PK-resistant PrP^Sc^ in VPSPr, sCJD and GSS. Purple rectangle represents the full-length (FL)-PrP23–231 with the GPI anchor (blue circular arrow). Gray rectangles: sCJDT1: sCJD PrP^Sc^ type 1 with an N-terminal starting residue 82 [45]; sCJDT2: sCJD PrP^Sc^ type 2 with an N-terminal starting residue 97 [45]; sCJD-CTF13: sCJD PrP^Sc^ C-terminal fragment with an N-terminal starting residue 154 [9]; sCJD-CTF12: sCJD PrP^Sc^ C-terminal fragment with an N-terminal starting residue 162 [9]. T1AF: Anchorless fragment of PrP^Sc^ type 1 with an unknown C-terminal truncation site (…) [46]; T2AF: Anchorless fragment of PrP^Sc^ type 2 with an unknown C-terminal truncation site (…) [46]. Yellow rectangle: GSS PrP^Sc^ fragment PrP7–8 with a N-terminal starting residue 82 and a C-terminal ending residue 153 from [43]. Green rectangles represent the 1E4-detected VPSPr PrP^Sc^ fragments with anticipated N-terminal starting at residue 86, but different C-termini [14] (current study): VPSPr-N20 with an intact GPI anchor, VPSPr-N17 with a molecular weight of 17 kDa but an unknown C-terminal truncation site (…), and VPSPr-N7 with an anticipated C-terminal ending residue 145. Red rectangles represent the anti-C antibody-detected VPSPr PrP^res^ fragments with anticipated intact C-termini carrying GPI anchor [14] (current study): VPSPr-C20 with an N-terminal starting residue 86, VPSPr-C18 with an anticipated N-terminal truncation site at residue 104, VPSPr-C12/13 with an anticipated N-terminal truncation site at residue 158, and VPSPr-C8–9 with an anticipated N-terminal truncation site at residue 190.

In the present study, we made several new findings about the unique prions (Figure 8, Supplemental Table S1). First, formation of the peculiar ladder-like PrP^res^ in VPSPr is a PK dose-dependent two-step process, which can be enhanced by basic pH. Second, two sets of PrP^res^ fragments in VPSPr can be identified with 1E4 against PrP97–105 and anti-C against PrP220–231. Third, two additional antibodies directed against PrP regions similar to 1E4 and anti-C epitopes are also able to recognize the two sets of PrP^Sc^ fragments. Fourth, while 1E4 shows the highest affinity to VPSPr7, 12B2 with an epitope against PrP90–94 has the highest affinity to GSS PrP7–8 among the PrP-specific antibodies examined. Fifth, PrP^P102L^ mutation in GSS virtually completely eliminates the affinity of 1E4 to the PrP^P102L^ mutant. Sixth, antibodies directed against specific PrP glycoforms show that migration of PrP Mono181 or Mono197 in VPSPr is faster than that of related PrP glycoforms in sCJD or non-CJD controls. Finally, although using different substrates and compared to different sCJD subtypes, our RT-QuIC results were consistent with the previous observations [24,44] that PrP^Sc^-seeding activity is lower and its lag time is longer in VPSPr than in sCJD and GSS^P102L^. Our findings raise several important issues and implications as to the molecular mechanism underlying the formation of this peculiar ladder-like electrophoretic profile of PK-induced N-terminally, or intermediately truncated multiple PrP^res^ fragments, and transmissibility of the disease.

Both homogenate pH and PK concentration have been reported to affect PK-induced truncation of PrP^Sc^ of sCJD. For instance, there were multiple unglycosylated PrP^res^ fragments detectable at PK of 100 µg/mL with highest intensity gradually shifting from 23 kDa to 20 kDa upon increasing pH from 5.0 to 7.1, which finally became a single band migrating at ~20 kDa over increasing brain homogenate pH to 7.2 and remained stable until pH 8.0 [35]. The same shifting pattern was also observed when increasing PK concentration from 7 to 100 µg/mL at pH 8.0 [35]. The function of PK is believed to be more efficient at basic pH so that it helps generate a stable PrP^Sc^ core fragment. However, in the case of VPSPr, PK-digestion of PrP^Sc^ at pH 8.0 generated a more prominent ladder-like electrophoretic profile of 5 PrP^res^ fragments. With PK digestion at pH 7.4 in standard lysis buffer, 3F4 showed two PrP^res^ fragments corresponding to Mono197 (VPSPr26) and unglycosylated PrP (VPSPr20) in VPSPr129MM and VPSPr129 MV; 1E4 did not clearly display VPSPr23, 17 and 7 as intense as VPSPr26 and 20. In contrast, at pH 8.0 in lysis buffer plus, 3F4 was able to detect two additional PrP^res^ including VPSPr23 and 17 besides VPSPr26 and 20 in Met-involved genotypes; 1E4 also revealed more intense VPSPr23, 17 and 7. Western blotting of PrP treated with different amounts of PK revealed that VPSPr23 may derive from C-terminal truncation of VPSPr26, VPSPr17 from C-terminal truncation of VPSPr20, and VPSPr7 probably from both VPSPr20 and 17 (Figure 1). Consistent with this assumption, the intensity of VPSPr26 and 20 was decreased, following increases in the intensity of VPSPr23, 17, and 7 over increasing PK concentrations until 50 µg/mL. PNGase F treatment after PK-digestion exhibited that VPSPr20, 17 and 7 are the core PrP^res^; moreover, the intensity of VPSPr20 decreased while the intensity of VPSPr17 and 7 was increased over an increase in the PK concentrations until 200 µg/mL (Figure 6). All these results indicate that generation of the peculiar ladder-like electrophoretic profile of PrP^res^ is a PK dose-dependent two-step process, which is boosted by basic pH 8.0, suggesting that PrP^Sc^ from VPSPr has a conformation that has never been seen in any sporadic human prion diseases before.

Another significant feature of VPSPr PrP^Sc^ is its higher affinity to 1E4 and lower affinity to 3F4, although the epitopes of the two antibodies are localized next to each other within the protein (Figure 2) [13,14,27,28]. 1E4 is the monoclonal antibody that recognizes an insoluble and PK-resistant PrP species termed iPrP^C^ in non-CJD brain homogenates and uninfected cultured cell lysates [16,17,34,47,48]. Our current 2D study further observed that 1E4 has a poorer affinity to the full-length PrP and a higher affinity to the truncated PrP, indicating that its epitope is tightly concealed in the full-length protein even after strong denaturation, reduction, and alkylation of treatment with 2D solutions containing dithiothreitol, iodoacetamide and a high concentration of urea. Deglycosylation also does not increase its affinity, suggesting that the low accessibility of the 1E4 epitope in the full-length PrP is determined by the backbone of the protein. This is in agreement with findings that the affinity of the 1E4 to the VPSPr7 is highest among the five VPSPr PrP^res^ fragments. So, it is most likely that both neighboring N- and C-terminal areas of the 1E4 epitope may be associated with this concealing effect.

Our previous study demonstrated that 1E4 is able to detect three intermediate PrP^res^ core fragments while anti-C detects four C-terminal core fragments after deglycosylation [14]. When compared the two sets of PrP^res^ fragments detected either by 1E4 or by anti-C, we noticed that only the top bands share the same molecular weight migrating at approximately 20 kDa, suggesting that it could be the same fragment containing both epitopes (Figure 8). Based on the molecular weight, locations of the two epitopes, and inclusion of possible GPI anchor with a molecular weight of ~4 kDa [9], we expect that the N-terminal starting amino acid could be residue 86. With this scenario, the compositions of the four core PrP^res^ detected by anti-C are anticipated to be: PrP86–231 for ~20 kDa fragment, PrP104–231 for ~18 kDa, PrP158–231 for ~12 kDa, and PrP190–231 for ~8–9 kDa. On the other hand, the 3 core PrP^res^ detected by 1E4 should be PrP86–231 for VPSPr20 and PrP86–145 for VPSPr7. However, there is difficulty in anticipating the sequence of VPSPr17 with the current scenario, including the residue 86 as the N-terminal starting amino acid and an intact GPI anchor. This is because, based on the molecular weight of VPSPr17, it should contain 154 amino acids (total molecular weight of the fragment/each amino acid molecular weight (17,000 Da/110 Da)). If it has the same N-terminal starting site from residue 86, it will be longer than 231 (86 + 154), which does not fit this scenario unless it has a different N-terminal starting site. To differentiate the two sets of fragments in description, we termed the 1E4-detected N-terminal core fragments as VPSPr-N20, N17, and N7 while the anti-C antibody-detected C-terminal core fragments as VPSPr-C20, C18, C12/13, and C8–9 (Figure 8). Further study with mass spectrometry is warranted to dissect the exact sequences of these PrP^res^ fragments.

The current study identified two additional antibodies that are able to detect the two sets of PrP^res^ fragments in VPSPr. T2, that has an epitope localized from PrP97–103 with the same N-terminal starting amino acid as 1E4 (Figure 2), recognized all five intermediate VPSPr PrP^res^ fragments (Figure 3C). EP1802Y, that has an epitope localized from PrP217–226 partially overlapping with the anti-C epitope (Figure 2), detected all C-terminal PrP^res^ fragments, as does anti-C (Figure 3D,E). Notably, 3F4 and 9A2 were able to weakly detect the top 4 VPSPr PrP^res^ fragments, but not VPSPr-N7. Although the exact reasons why they are able to detect all VPSPr PrP^res^ except for VPSPr-N7 remain to be determined, the possibility cannot be excluded that the N-terminal starting amino acid may be different between VPSPr-N7 and other VPSPr PrP^res^ fragments. Moreover, 9A2 also detected VPSPr-N7 but only at PK of 25 µg/mL or lower, whereas 1E4 recognized it mainly at PK of 25–50 µg/mL or higher. Its intensity was enhanced over the increase in the PK concentration. Given the above discrepancy in matching the N-terminal starting amino acid, it is possible that the N-terminal starting amino acids could be different among VPSPr PrP^res^ fragments although they are detected by 1E4 and T2 equally well.

Since GSS is also characterized by the presence of multiple small PrP^res^ fragments, VPSPr has been proposed to be more akin to subtypes of GSS [14,32,33]. The PrP7–8 from GSS^P102L^ is an intermediate PrP^res^ with both ragged N-termini starting at residues from 74 to 82 and ragged C-termini ending at residues 147–153; it is detectable by western blotting with 3F4 although its level was highly variable [43]. Our GSS^P102L^-129M case exhibited no PrP7–8 in the samples treated with PK alone and only showed a faint PrP7–8 band in the samples treated with both PK and PNGase F probing with 3F4, which could result from the smaller amount. The epitopes of antibodies examined including 12B2, 1E4, 9A2, and 3F4 are all within the PrP7–8 sequence (Figure 2). However, only 12B2 was able to detect this fragment highly efficiently. Notably, although this antibody also recognized VPSPr-N26 and N20, it was unable to detect VPSPr-N23, N17, and N7. Interestingly, we also observed that 1E4 had the poorest affinity to PrP from GSS^P102L^ compared to other antibodies, which could be due to the PrP^P102L^ mutation that falls within the epitope and disrupts the binding of 1E4 to the mutant PrP.

Our previous results suggested that the inability to convert diglycosylated and Mono181 PrP into PrP^Sc^ may contribute to altered PrP glycosylation in VPSPr [27,28]. In this study, we showed that the banding pattern of Mono181 and Mono197 is obviously different between VPSPr and sCJD or non-CJD. Compared to those of sCJD or non-CJD, the Mono181 and Mono197 in VPSPr were faint and thin and their migrations were faster than those in sCJD or non-CJD. The difference in the migration of the two bands between VPSPr and sCJD or non-CJD became smaller after deglycosylation with PNGase F, suggesting that the difference may arise mainly from abnormal glycosylation in VPSPr. The changed glycans at residue 181 could alter local conformation around residue 181, which is close to the β-sheets 2/α-helixes 2 loop, the critical region implicated in dominant-negative inhibition [49]. Indeed, there appear to be significant differences in the effect of mutations occurring at residue 181 or 197 on the conversion of PrP^C^ into PrP^Sc^. For instance, while most of the mutations at residue 181 blocked the conversion, no mutations at residue 197 blocked PrP^C^ conversion in cell and animal models [50,51]. More specifically, interactions between different PrP^C^ glycoforms have been found to mediate the efficiency of prion formation, involving glycan-associated steric hindrance [52]. Given that no PrP mutations have been observed in VPSPr, the possibility cannot be excluded that a similar abnormal glycosylation at residue 181 caused by a rare stochastic event may trigger the pathological processes as described for fCJD^V180I^ [27].

We found that the seeding activity of PrP^Sc^ was detectable in all three genotypes of VPSPr, although lower than that of sCJDMV2 and GSS^P102L^, by RT-QuIC, which is consistent with previous studies, regardless of different substrates and comparing with different sCJD subtypes [24,44]. Not only were the prion ThT fluorescence intensities lower in reactions seeded with VPSPr compared to sCJD, but the lag times of aggregation were longer. According to Log seeding dose 50 (SD50) from our RT-QuIC assay, the seeding activity of VPSPr (6.7) was lower than that of sCJD (8.6) and GSS^P102L^ (7.7) but higher than that of fCJD^V180I^ (5.7). Among the three genotypes of VPSPr, the seeding activity of VPSPr129VV was highest (7.1), followed by VPSPr129MM (7.0) and VPSPrMV (6.1). It is worth noting that while VPSPr129VV showed the least amount of PrP^res^, this condition was observed to have the highest prion-seeding activity among the three genotypes by RT-QuIC. As we revealed previously, VPSPr129VV has the highest amount of PK-sensitive PrP^Sc^ [14]. The possibility cannot be ruled out that the prion seeding activity detected by RT-QuIC may be mainly associated with PK-sensitive rather than PK-resistant PrP^Sc^. This possibility warrants further study in the future. The lower PrP^Sc^-seeding activity appears to be in agreement with its lower transmissibility shown by animal transmission studies in humanized transgenic (Tg) mice and bank voles [29,30]. In studies with humanized Tg mice, no clinical signs were detected although a few small immature-like plaques could be found in the brain of infected mice [29,30]; however, such transmission of aggregates was no longer detectable in the second passage [29]. Moreover, although a lower transmission rate of the disease could be found in the first passage of infected bank voles, no typical VPSPr-like PrP^Sc^ was detected in the brain of infected bank voles [31].

In summary, PrP^Sc^ from VPSPr is characterized by many new biochemical and biological properties. Our current study provides new evidence to favor the hypothesis that VPSPr is associated with a new prion strain that has never been seen in any other human prion diseases. The disease may bear a new pathogenesis involving a deficiency in glycosylation. This deficiency may lead to a higher sensitivity to PK-digestion and lower seeding activity/infectivity of the protein so that it has smaller amounts of PrP^Sc^ conformers and is vulnerable to truncations, to form multiple small fragments by PK-digestion.

## 4. Materials and Methods

### 4.1. Reagents and Antibodies

Proteinase K (PK) was purchased from Sigma (St. Louis, MO, USA). Protease inhibition cocktail tablets were from Roche Diagnostics (Indianapolis, IN, USA). Reagents for enhanced chemiluminescence (ECL Plus) were purchased from Thermo Scientific (Rockford, IL, USA). PrP-specific antibodies used in this study (Figure 2) included mouse monoclonal antibody mAb 3F4 against PrP106–112 [15,39], mAb 1E4 against PrP97–105 [16], polyclonal antibody (pAb) Tohoku 2 (T2) against PrP97–103 [37], pAb EP1802Y against PrP217–226 [38], pAb anti-C antibody recognizing the C-terminal PrP220–231 [9], mAb 9A2 against PrP99–101 [40], mAb 12B2 against PrP90–94 [40], and pAb anti-N against PrP23–40 [41]. V14 and Bar209 mAbs recognize PrP^C^ mono-glycosylated on the first (V14) (PrP168–181) or the second (Bar209) (PrP185–196) glycosylation sites, respectively, as well as unglycosylated forms [42,53,54]. Sheep anti-mouse IgG and donkey anti-rabbit IgG conjugated with horseradish peroxidase were purchased from Sigma Aldrich and used as secondary antibodies for the primary mAbs and pAbs, respectively [47].

### 4.2. Preparation of Brain Samples

Post-mortem brain tissues of cadavers with sCJD (1 case with sCJDMM1, 1 with sCJDMM2, and 2 with sCJDMV2), VPSPr (2 with VPSPr129MM, 3 with VPSPr129VV, and 4 with VPSPr129MV), GSS linked to PrP^P102L^ mutation (GSS^P102L^, *n* = 3), familial CJD linked to PrP^V180I^ mutation (fCJD^V180I^ with 129MM, *n* = 3), or non-CJD controls (1 with PrP-129MM and 1 with PrP-129VV) were collected through the National Prion Disease Pathology Surveillance Center, Department of Pathology, Case Western Reserve University School of Medicine, Cleveland, Ohio. Lysis buffer ‘standard’ (100 mM NaCl, 10 mM EDTA, 0.5% NP-40, 0.5% sodium deoxycholate, 10 mM Tris-HCl, pH 7.4) or lysis buffer ‘plus’ (100 mM NaCl, 10 mM EDTA, 0.5% NP-40, 0.5% sodium deoxycholate, 100 mM Tris-HCl, pH 8.0) was used [35,36]. Human brain homogenates were prepared in lysis buffer ‘standard’ or ‘plus’ at 10% (*w*/*v*), by homogenization in a Mini-Beadbeater (BioSpec, Laboratory Supply Network, Inc., Atkinson, NH, USA) 3 times within 1 min. When required, brain homogenates were centrifuged at 1000× *g* for 10 min at 4 °C to collect supernatant (S1). To prepare detergent-soluble (S2) and -insoluble fractions (P2), S1 fractions were further centrifuged at 35,000 rpm (100,000× *g*) in an SW55 rotor (Beckman Coulter, Fullerton, CA, USA) at 4 °C for 1 h. After ultracentrifugation, the supernatants that contain S2 were transferred into clean tubes. The pellets that contain the P2 fractions were further resuspended in the lysis buffer as previously described [47].

### 4.3. RT-QuIC Assay

RT-QuIC assays of brain samples from various human prion diseases were conducted as previously described [44,55,56] with minor modification. In brief, RT-QuIC reaction mix was composed of 1 x phosphate buffer, pH 7.4, 0.17M NaCl, 0.1 mg/mL recombinant truncated Syrian golden hamster PrP90–231, 10 µM Thioflavin T (ThT), 1 mM EDTA, and 0.001% SDS. Aliquots of the reaction mix (98 µL) were loaded into each well of a 96-well plate (Nunc) and seeded with 2 µL of brain homogenate at a final concentration of 10^−3^ through 10^−9^ as designated. Brain homogenate seeds were spun at 5000× *g* at 4 °C for 5 min to remove any debris prior to loading. The plates were sealed with a plate sealer film (Nalgene Nunc International), placed in a BMG FLUOstar Omega plate reader and incubated at 42 °C with cycles of 1 min shaking (700 rpm double orbital) and 1 min rest throughout the indicated incubation times. ThT fluorescence intensity (450 ± 10 nm excitation and 480 ± 10 nm emission; bottom read) was determined every 45 min. Each sample was run in quadruplicate and the average fluorescence values per sample was calculated based on all four replicate wells regardless of whether their values crossed the determined positive threshold or not as previously described [44,55,56]. At least 2 of 4 replicates were required to cross the threshold for a sample to be considered as positive.

### 4.4. Two-Dimensional Western Blotting

Two-dimensional (2D) western blotting of PrP was performed as previously described [47]. Briefly, tissue homogenates were boiled in SDS sample buffer (3% SDS, 2 mM EDTA, 4% β-mercapto-ethanol, 10% glycerol, 50 mM Tris, pH 6.8), followed by precipitation with 5 volumes of pre-chilled methanol at −20 °C for 2 h, then centrifugation at 14,000 rpm at 4 °C for 30 min. The pellets were resuspended in 50 µL reducing buffer (8 M urea, 2% CHAPS, 5 mM tributyl-phosphine, 20 mM Tris, pH 8.0) at room temperature (RT) for 1 h, then added 5 µL iodoacetimate (200 mM) in dark at RT for more than 1 h. Five volumes of pre-chilled methanol were added and incubated at −20 °C for 2 h and centrifuged at 14,000 rpm at 4 °C for 30 min. The pellets were resuspended in 200 µL of rehydration buffer (7 M urea, 2 M Thio-urea, 1% DTT, 1% CHAPS, 1% Triton X-100, 1% ampholyte pH 3–10, trace of amount bromophenol blue) and centrifuged at 5000 rpm for 5 min at RT.

Samples were then loaded onto the immobilized pH gradient strips for rehydration at RT for more than 12 h with gentle shaking. The first dimensional isoelectric focusing was performed on the rehydrated gel strips for 7 h using a focusing tray having undergone 4 stages from low voltage for 15 min and to the maximal voltage of 8000 V for the remainder of the time. For the second dimension SDS-PAGE, the focused gel strips were equilibrated for 15 min each in equilibration buffer A (6 M urea, 2% SDS, 20% glycerol, 130 mM dithiothreitol, 0.375 M Tris-HCl, pH 8.8) and equilibration buffer B (6 M urea, 2% SDS, 20% glycerol, 135 mM iodoacetamide, 0.375 M Tris-HCl, pH 8.8), respectively. The equilibrated strips were loaded onto10–20% Criterion™ Tris-HCl Protein Gel at 150 V for 120 min. The remaining steps followed traditional western blotting procedures as described below.

### 4.5. Western Blotting

To determine PrP^res^ fragment patterns, samples were incubated with the designated PK concentration for an hour at 37 °C with shaking at 450 rpm. After incubation, protease inhibitor cocktail (Roche) and SDS sample buffer were added prior to boiling for 10 min to terminate the PK reaction. Samples without PK treatment were directly added to SDS sample buffer to detect untreated PrP. Samples were loaded onto 15% Tris-HCl Criterion pre-cast gels (Bio-Rad) for SDS-PAGE. After SDS-PAGE, the proteins on the gels were transferred to Immobilon-P Polyvinylidene Difluoride (PVDF, Millipore) for 90 min at 0.35 A. After blocking in 5% milk in 0.1% tween-20 TBS (TBST) washing buffer for one hour at RT, the membranes were incubated at RT with designated PrP-specific antibodies overnight. The membranes were washed with TBST for 5 min for 4 times, then incubated with HRP-conjugated sheep anti-mouse or donkey anti-rabbit IgG. The protein bands or spots were visualized on Kodak film by ECL Plus following the product instruction.

### 4.6. Statistical Analysis

The differences in ThT fluorescence and lag time of PrP aggregation-seeding activity among different groups were statistically analyzed using Student’s t-test to obtain *p* values. All tests adopted a two-sided type I error level of 0.05.

## Data Availability

All materials used in this study will be made available subject to a material transfer agreement.

## Supplementary Materials

The following are available online at https://www.mdpi.com/article/10.3390/pathogens10050513/s1, Table S1: Title: Summary of findings made in this study.

## Abbreviations

PrP^C^: cellular prion protein; PrP^Sc^: scrapie isoform of prion protein; PK: proteinase K; PrP^res^: PK-resistant PrP^Sc^; VPSPr: variably protease-sensitive prionopathy; vCJD: variant Creutzfeldt-Jakob disease; sCJD: sporadic Creutzfeldt-Jakob disease; fCJD^V180I^: familial Creutzfeldt-Jakob disease linked to PrP^V180I^ mutation; GSS: Gerstmann-Sträussler-Scheinker disease; RT-QuIC: Real-time quaking-induced conversion; 2D: two-dimensional electrophoresis; T2: Tohoku-2; FL: full-length prion protein; C1: C-terminal fragment truncated at residue 111 or 112; C2: C-terminal fragment truncated at approximately residue 90.
