# Supplementary material for: Further Characterization of Glycoform-Selective Prions of Variably Protease-Sensitive Prionopathy
Source: Pathogens. 2021 Apr 23;10(5):513. doi: 10.3390/pathogens10050513 (PMC8146342; doi:10.3390/pathogens10050513)
Supplement: Supplementary file 1 [file pathogens-10-00513-s001.zip › pathogens-1177674-supplementary.pdf]

Further Characterization of Glycoform-selective Prions of Variably Protease-Sensitive Prionopathy

Weiguanliu Zhang[1,2], Xiangzhu Xiao[2], Mingxuan Ding[1,2], Jue Yuan[2], Aaron Foutz[2], Mohammed Moudjou[3], Tetsuyuki Kitamoto[4], Jan Langeveld[5], Li Cui[1§], Wen-Quan Zou[2,6§]

[1]Department of Neurology, The First Hospital of Jilin University, Changchun, Jilin Province, China.

[2]Department of Pathology, Case Western Reserve University School of Medicine, Cleveland, Ohio 44106, USA.

[3]Université Paris Saclay, INRAE, UVSQ, VIM, Jouy-en-Josas, France.

[4]Department of Neurological Science, Tohoku University Graduate School of Medicine, 2-1 Seiryo-machi, Aoba-ku, Sendai, Miyagi 980-8575, Japan.

[5]Department of Infection Biology, Wageningen Bioveterinary Research, 12 8221RA 39 Lelystad, The Netherlands.

[6]National Prion Disease Pathology Surveillance Center, Case Western Reserve University School of Medicine, Cleveland, Ohio 44106, USA.

§Corresponding authors: Wen-Quan Zou: wxz6@case.edu; Li Cui: chuili1967@126.com

Supplementary Table S1

Table S1 Summary of findings made in this study

Measurements / PrP^res fragments			Antibody mapping: 1E4 97-105	3F4 106-112	9A2 99-101	12B2 90-94	V14 168-181	Bar209 185-196	Anti-C 220-231	Tohoku 2 97-103	EP1802Y 217-226	RT-QuIC assay: Seeding activities (SD_{50})	Lag Time (h)
sCJD PrP^res	Types	T1 (82-231)	-/+*	+¶	+	+	+	+	+	-§	-	8.6	4.3
		T2 (97-231)	+	+	+	-	+	+	+	+	+		
	sCJD-CTF13 (154-231)		-	-	-	-	+	+	+	-	+		
	sCJD-CTF12 (162-231)		-	-	-	-	+	+	+	-	+		
GSS^P102L PrP7-8 (82-153)			-	+	+	+	n/d**	n/d	-	n/d	-	7.7	26.0
VPSPr PrP^res	pH 7.4	VPSPr-N20 (86-231)	+	-/+	+	-/+	n/d	n/d	+	+	+	VPSPr 129MM: 7.0	23.1
		VPSPr-C18 (104-231)	-	-	-	-	+	+	+	-	+		
		VPSPr-N17 (86-?)	+	-/+	+	-	n/d	n/d	-	+	-		
		VPSPr-C12/13 (158-231)	-	-	-	-	+	+	+	-	+		
		VPSPr-C8-9 (190-231)	-	-	-	-	-	n/d	+	-	+	VPSPr 129MV: 6.1	31.1
		VPSPr-N7 (86-145)	+	- (+ in 2D)	-/+	-	-	-	-	+	-		
	pH 8.0	VPSPr-N20 (86-231)	+	+	n/d	n/d	n/d	n/d	n/d	n/d	n/d		
		VPSPr-C18 (104-231)	-	-	n/d	n/d	n/d	n/d	n/d	n/d	n/d		
		VPSPr-N17 (86-?)	+	+	n/d	n/d	n/d	n/d	n/d	n/d	n/d	VPSPr 129VV: 7.1	18.0
		VPSPr-C12/13 (158-231)	-	-	n/d	n/d	n/d	n/d	n/d	n/d	n/d		
		VPSPr-C8-9(190-231)	-	-	n/d	n/d	n/d	n/d	n/d	n/d	n/d		
		VPSPr-N7 (86-145)	+	-	n/d	n/d	n/d	n/d	n/d	n/d	n/d		
fCJD^V180I			+	-/+	n/d	n/d	n/d	n/d	n/d	n/d	n/d	5.7	36.7

Note: *: Weak reaction; ¶: positive reaction; §: no reaction; **: not detected in this study.
